# Nicotine Pouch Use in Youths and Adults Who Use Cigarettes, E-Cigarettes, and Smokeless Tobacco

**DOI:** 10.1001/jamanetworkopen.2025.11630

**Published:** 2025-05-12

**Authors:** Amanda M. Palmer, Tracy T. Smith, Andrew A. Chen, Alana M. Rojewski, Matthew J. Carpenter, Benjamin A. Toll

**Affiliations:** 1 Department of Public Health Sciences, Medical University of South Carolina, Charleston; 2 Hollings Cancer Center, Medical University of South Carolina, Charleston; 3 Department of Psychiatry and Behavioral Sciences, Medical University of South Carolina, Charleston

## Abstract

This cross-sectional study describes nicotine pouch use among adolescents and adults who use other nicotine products.

## Introduction

The tobacco marketplace is shifting such that noncombustible (ie, less harmful) products are displacing more harmful combustible products. One product gaining traction is tobacco-free oral nicotine pouches, which are small, permeable pouches containing a synthetically-produced, crystalized nicotine salt powder (and stabilizers, fillers, pH adjusters, and sweeteners).^1^ Market analyses show exponential growth in the sales of these products, which is projected to continue to increase, possibly due to rapid delivery of high nicotine concentrations, increased marketing, and appealing flavors.^2^ This study provides an estimate of nicotine pouch use prevalence, inclusive of other tobacco use history, among youths and adults in the US.

## Methods

This cross-sectional study was exempt from review per 45 CFR §46.104. All participants provided informed consent.^3^ This study is reported following the Strengthening the Reporting of Observational Studies in Epidemiology (STROBE) reporting guideline. Data for this study were collected as a part of the Population Assessment of Tobacco and Health (PATH)^4^ wave 7 (January 2022 to April 2023) youths (age 12-17 years; response rate, 54.3%) and adult (age ≥18 years; response rate, 52.4%) cohorts, a nationally representative, publicly available longitudinal US survey approved by the Westat institutional review board. Race and ethnicity were self-reported; further details are provided in the eMethods in Supplement 1.

Analyses present pouch use outcomes by demographics and other relevant tobacco use characteristics (eg, smoking history). Pouch outcomes of interest include ever use, past 12 months use, and past 30 days use. Balanced repeated replication weighting procedures were used for frequency analyses, completed on SAS software version 9.4 (SAS Institute) from January to March 2025. Logistic regression models estimated the association of sociodemographic variables on outcomes, adjusted for all other demographics, and tobacco use variables on outcomes, adjusted for demographics, with a 2-sided Bonferroni corrected α = .0167.

## Results

The analytic sample included 10 632 adolescents (51.25% [95% CI, 51.18%-51.33%] male; 25.82% [95% CI, 25.68%-25.95%] Hispanic; 65.25% [95% CI, 64.71%-65.80%] White) and 29 754 adults (48.48% [95%CI, 48.44%-48.51%] male; 17.14% [95% CI, 17.08%-17.21%] Hispanic; 74.40% [95%CI, 74.07%-74.74%] White) (eMethods in Supplement 1). As shown in Table 1 and Table 2, 0.85% (95% CI, 0.69%-1.01%) of adolescents and 3.34% (95% CI, 3.04%-3.64%) of adults reported ever using oral nicotine pouches, 0.60% (95% CI, 0.47%-0.73%) of adolescents and 1.58% (95% CI, 1.39%-1.77%) of adults reported past-year use, and 0.21% (95% CI, 0.13%-0.30%) of adolescents and 0.82% (95% CI, 0.69%-0.96%) of adults reported use in the past 30 days. Older adolescents had higher odds of pouch use at each time point (ever, past 12 months, and past 30 days) relative to their younger counterparts, whereas younger adult age groups had higher odds of pouch use relative to those older than 65 years. In both cohorts, odds of pouch use were higher at each time point for males than females. Within both cohorts, individuals with current or former cigarette, e-cigarette, or smokeless tobacco use history had higher odds of pouch use at each time point relative to those without a tobacco use history. Finally, odds of pouch use were higher at each time point among individuals who had used other oral tobacco or cannabis in the past year compared with those who had not.

**Table 1.** Nicotine Pouch Use Among US Adolescents

Characteristic	Ever				Past 12 mo					Past 30 d				
	No. (%)^a^	Weighted, % (95% CI)^b^	aOR (95% CI)^c^	*P* value^d^	No. (%)^a^	Weighted, % (95% CI)^b^	aOR (95% CI)^c^	*P* value^d^	No. (%)^a^		Weighted, % (95% CI)^b^	aOR (95% CI)^c^	*P* value^d^
Overall (n = 10 632), No.	105	0.85 (0.69-1.01)	NA	NA	75	0.60 (0.47-0.73)	NA	NA	29		0.21 (0.13-0.30)	NA	NA
Sex													
Male	77 (73.33)	1.20 (0.95-1.46)	2.65 (1.69-4.17)	<.001	58 (77.33)	0.90 (0.67-1.14)	3.46 (1.95-6.15)	<.001	27 (93.10)		0.39 (0.24-0.55)	24.36 (3.30-179.72)	.002
Female	27 (25.71)	0.47 (0.29-0.66)	1 [Reference]	NA	16 (21.33)	0.27 (0.13-0.42)	1 [Reference]	NA	2 (6.90)		0.02 (0.00-0.06)	1 [Reference]	NA
Age, y													
12-14	21 (20.00)	0.39 (0.21-0.57)	1 [Reference]	NA	10 (13.33)	0.19 (0.08-0.31)	1 [Reference]	NA	4 (13.79)		0.07 (0.00-0.14)	1 [Reference]	NA
15-17	84 (80.00)	1.33 (1.03-1.63)	3.35 (2.05-5.49)	<.001	65 (86.67)	1.02 (0.75-1.30)	5.08 (2.59-9.94)	<.001	25 (86.21)		0.36 (0.20-0.52)	4.85 (1.67-14.05)	.004
Race													
Black	1 (<1)	0.02 (0.00-0.06)	0.05 (0.01-0.38)	.004	1 (1.33)	0.02 (0.00-0.06)	0.08 (0.01-0.57)	.0118	1 (3.45)		0.02 (0.00-0.06)	0.24 (0.03-1.83)	.17
White	90 (85.71)	1.20 (0.95-1.44)	1 [Reference]	NA	65 (86.67)	0.86 (0.65-1.06)	1 [Reference]	NA	25 (86.21)		0.31 (0.18-0.44)	1 [Reference]	NA
Other^e^	14 (13.33)	0.56 (0.31-0.82)	0.68 (0.38-1.22)	.20	9 (12.00)	0.36 (0.15-0.56)	0.60 (0.29-1.26)	.18	3 (10.34)		0.11 (0.00-0.25)	0.64 (0.19-2.13)	.46
Ethnicity													
Hispanic	10 (9.52)	0.32 (0.10-0.54)	3.72 (1.84-7.53)	<.001	6 (8.00)	0.18 (0.03-0.33)	4.35 (1.72-11.04)	.002	1 (3.45)		0.01 (0.00-0.04)	8.09 (1.07-60.93)	.04
Non-Hispanic	92 (87.62)	1.04 (0.83-1.25)	1 [Reference]	NA	67 (89.33)	0.75 (0.59-0.92)	1 [Reference]	NA	27 (93.10)		0.2 (0.17-0.40)	1 [Reference]	NA
Income, $													
<50 000	24 (22.86)	0.61 (0.36-0.86)	0.98 (0.61-1.58)	.94	13 (17.33)	0.33 (0.13-0.53)	0.72 (0.38-1.33)	.29	4 (13.79)		0.07 (0.00-0.15)	0.56 (0.19-1.65)	.29
≥50 000	79 (75.24)	1.02 (0.77-1.27)	1 [Reference]	NA	60 (90.00)	0.77 (0.55-0.99)	1 [Reference]	NA	24 (82.76)		0.29 (0.16-0.42)	1 [Reference]	NA
Cigarette use^f^													
Current	19 (18.10)	14.17 (8.38-19.98)	38.82 (20.45-73.68)	<.001	14 (18.67)	10.60 (5.26-15.94)	33.59 (16.06-70.22)	<.001	9 (31.03)		5.14 (1.06-9.22)	59.82 (21.53-166.17)	<.001
Former	49 (46.67)	7.39 (5.47-9.31)	17.74 (11.13-28.30)	<.001	33 (44.00)	4.93 (3.29-6.56)	14.78 (8.54-25.59)	<.001	11 (37.93)		1.45 (0.72-2.19)	13.31 (5.12-34.56)	<.001
Never	37 (35.24)	0.33 (0.21-0.45)	1 [Reference]	NA	28 (37.33)	0.24 (0.14-0.34)	1 [Reference]	NA	9 (31.03)		0.07 (0.03-0.15)	1 [Reference]	NA
E-cigarette use^g^													
Current	55 (52.38)	8.27 (5.60-10.65	57.87 (29.91-111.95)	<.001	41 (54.67)	6.09 (4.24-7.95)	48.48 (22.94-102.46)	<.001	24 (82.76)		3.24 (1.83-4.66)	99.46 (27.89-354.71)	<.001
Former	36 (34.29)	3.17 (2.17-4.18)	20.10 (10.34-39.10)	<.001	23 (30.67)	2.06 (1.24-2.88)	14.86 (6.88-32.10)	<.001	2 (6.90)		0.15 (0.00-0.37)	5.43 (0.84-34.99)	.08
Never	14 (13.33)	0.14 (0.05-0.22)	1 [Reference]	NA	11 (14.67)	0.10 (0.03-0.17)	1 [Reference]	NA	3 (10.34)		0.03 (0.00-0.06)	1 [Reference]	NA
Smokeless tobacco use^h^													
Current	10 (9.52)	50.12 (28.09-72.11)	72.37 (25.96-201.73)	<.001	10 (13.33)	50.10 (28.09-72.12)	89.61 (31.06-258.59)	<.001	7 (24.14)		34.23 (13.03-55.42)	148.03 (45.47-481.98)	<.001
Former	33 (31.43)	20.59 (14.26-26.91)	34.60 (20.92-57.22)	<.001	21 (28.00)	12.89 (7.34-18.43)	27.59 (15.15-50.22)	<.001	8 (27.59)		4.13 (0.94-7.31)	24.75 (9.24-66.29)	<.001
Never	62 (59.05)	0.53 (0.39-0.67)	1 [Reference]	NA	44 (58.67)	0.37 (0.26-0.48)	1 [Reference]	NA	14 (48.28)		0.11 (0.05-0.18)	1 [Reference]	NA
Other oral product use^i^													
Past 12 mo	15 (14.29)	27.82 (12.98-42.66)	56.26 (25.19-125.65)	<.001	15 (20.00)	27.82 (12.98-42.66)	96.11 (40.54-227.82)	<.001	5 (17.24)		8.06 (0.00-16.32)	51.10 (14.63-178.53)	<.001
None	89 (84.76)	0.72 (0.56-0.87)	1 [Reference]	NA	60 (80.00)	0.47 (0.36-0.60)	1 [Reference]	NA	24 (82.76)		0.18 (0.10-0.26)	1 [Reference]	NA
Cannabis use													
Past 12 mo	39 (37.14)	3.38 (1.90-4.86)	6.87 (3.872-12.19)	<.001	15 (20.00)	2.09 (1.02-3.17)	6.68 (3.30-13.54)	<.001	5 (17.24)		0.64 (0.00-1.28)	6.09 (1.778-0.86)	.004
None	22 (20.95)	0.45 (0.28-0.62)	1 [Reference]	NA	25 (33.33)	0.29 (0.15-0.43)	1 [Reference]	NA	9 (31.03)		0.10 (0.03-0.17)	1 [Reference]	NA

Abbreviations: aOR, adjusted odds ratio; NA, not applicable.

^a^ Unweighted number and column percentages. Numbers may not add up to full sample due to missing data. Models with cell sizes less than 10 should be interpreted with caution due to unreliable estimates.

^b^ Row percentage of participants responding affirmatively compared with those responding No.

^c^ Models with demographic exposure variables (sex, age, race, ethnicity, income, and education) are adjusted for the other sociodemographic variables, and each model of tobacco or cannabis use exposure variables is adjusted for all sociodemographic variables.

^d^ Significance was determined after Bonferroni adjustment for the 3 reported outcomes (ever use, past 12-month use, past 30-day use) at a threshold *P* < .0167.

^e^ The other category included Asian and other races, including multiracial.

^f^ Current smoking is defined as smoking a cigarette within the past 30 days; former smoking, having ever smoked a cigarette but not within the past 30 days; and never smoking, having never smoked a cigarette.

^g^ Current vaping is defined as using an e-cigarette within the past 30 days; former vaping, having ever used an e-cigarette but not within the past 30 days; and never vaping, having never used an e-cigarette.

^h^ Smokeless tobacco is inclusive of dip, chewing tobacco, smokeless tobacco pouches, moist snuff, and snus. Current smokeless use is defined as using smokeless tobacco within the past 30 days; former smokeless, having ever used smokeless but not within the past 30 days; and never smokeless, having never used smokeless tobacco.

^i^ Other oral tobacco products include nontherapeutic gum or lozenges, toothpicks, discs, dissolvables, and tablets.

**Table 2.** Nicotine Pouch Use Among US Adults

Characteristic	Ever				Past 12 mo				Past 30 d			
	No. (%)^a^	Weighted, % (95% CI)^b^	aOR (95% CI)^c^	*P* value^d^	No. (%)^a^	Weighted, % (95% CI)^b^	aOR (95% CI)^c^	*P* value^d^	No. (%)^a^	Weighted, % (95% CI)^b^	aOR (95% CI)^c^	*P* value^d^
Overall (n = 29 754), No.	1408	3.34 (3.04-3.64)	NA	NA	763	1.58 (1.39-1.77)	NA	NA	375	0.82 (0.69-0.96)	NA	NA
Sex												
Male	1032 (73.30)	5.40 (4.92-5.89)	3.20 (2.82-3.64)	<.001	586 (76.80)	2.60 (2.29-2.92)	3.81 (3.19-4.56)	<.001	306 (81.60)	1.39 (1.18-1.60)	5.03 (3.83-6.604	<.001
Female	376 (26.70)	1.40 (1.22-1.60)	1 [Reference]	NA	177 (23.20)	0.63 (0.50-0.76)	1 [Reference]	NA	69 (18.40)	0.29 (0.19-0.40)	1 [Reference]	NA
Age, y												
18-24	505 (35.87)	5.57 (5.01-6.13)	4.87 (3.51-6.76)	<.001	343 (44.95)	3.76 (3.30-4.23)	21.69 (10.16-46.30)	<.001	141 (37.60)	1.55 (1.26-1.84)	22.71 (7.13-72.28)	<.001
25-34	431 (30.61)	5.52 (4.68-6.36)	5.49 (3.98-7.58)	<.001	235 (30.80)	3.05 (2.48-3.63)	18.18 (8.53-38.76)	<.001	122 (32.53)	1.64 (1.27-2.02)	21.81 (6.91-68.88)	<.001
35-44	187 (13.28)	3.82 (3.02-4.63)	4.16 (2.95-5.87)	<.001	87 (11.40)	1.79 (1.28-2.31)	11.50 (5.27-25.08)	<.001	53 (14.13)	1.07 (0.72-1.41)	16.40 (5.07-53.03)	<.001
45-54	132 (9.38)	3.34 (2.61-4.09)	3.50 (2.45-5.02)	<.001	59 (7.73)	1.19 (.88-1.51)	10.15 (4.59-22.42)	<.001	37 (9.87)	0.69 (0.46-0.92)	15.15 (4.63-49.56)	<.001
55-64	103 (7.32)	2.03 (1.57-2.51)	2.27 (1.57-3.29)	<.001	32 (4.19)	0.61 (0.36-0.86)	4.30 (1.86-9.91)	<.001	19 (5.07)	0.35 (0.14-0.57)	5.58 (1.61-19.31)	.0066
≥65	50 (3.55)	1.01 (0.60-1.43)	1 [Reference]	NA	7 (<1)	0.10 (0.01-0.20)	1 [Reference]	NA	3 (<1)	0.05 (0.00-0.10)	1 [Reference]	NA
Race												
Black	84 (5.97)	1.38 (1.01-1.75)	0.21 (0.17-0.27)	<.001	34 (4.46)	0.51 (0.31-0.72)	0.17 (0.12-0.25)	<.001	18 (4.80)	0.29 (0.12-0.46)	0.22 (0.13-0.35)	<.001
White	1156 (82.10)	3.88 (3.48-4.28)	1 [Reference]	NA	635 (83.22)	1.86 (1.60-2.12)	1 [Reference]	NA	314 (83.73)	0.97 (0.79-1.15)	1 [Reference]	NA
Other^e^	150 (10.65)	2.65 (2.06-3.25)	0.64 (0.53-0.77)	<.001	78 (10.22)	1.19 (0.86-1.52)	0.56 (0.44-0.72)	<.001	35 (9.33)	0.57 (0.34-0.80)	0.54 (0.37-0.78)	.001
Ethnicity												
Hispanic	155 (11.01)	1.48 (1.09-1.87)	1 [Reference]	NA	82 (10.75)	0.68 (0.44-0.92)	1 [Reference]	NA	37 (9.87)	0.40 (0.20-0.60)	1 [Reference]	NA
Non-Hispanic	1231 (87.43)	3.74 (3.40-4.08)	2.90 (2.41-3.49)	<.001	671 (87.94)	1.79 (1.56-2.01)	3.21 (2.47-4.15)	<.001	333 (88.80)	0.92 (0.77-1.07)	3.13 (2.15-4.57)	<.001
Income, $												
<50 000	595 (42.26)	3.13 (2.76-3.48)	1.01 (0.89-1.14)	.89	308 (40.37)	1.42 (1.18-1.67)	1.02 (0.87-1.21)	.79	144 (38.40)	0.71 (0.55-0.86)	1.01 (0.80-1.27)	.95
≥50 000	751 (53.34)	3.62 (3.17-4.07)	1 [Reference]	NA	424 (55.57)	1.79 (1.52-2.06)	1 [Reference]	NA	222 (59.20)	0.98 (0.79-1.16)	1 [Reference]	NA
Education												
Less than HS	114 (8.10)	2.93 (2.30-3.56)	1.00 (0.76-1.32)	.98	44 (5.77)	0.99 (0.62-1.38)	0.64 (0.43-0.95)	.03	19 (5.07	0.46 (0.23-0.69)	0.48 (0.27-0.85)	.01
HS or GED	398 (28.27)	3.64 (3.13-4.15)	1.24 (1.03-1.48)	.02	207 (27.13)	1.60 (1.30-1.91)	0.91 (0.72-1.16)	.44	95 (25.33)	0.80 (0.55-1.05)	0.76 (0.55-1.06)	.10
Some college	545 (38.71)	4.20 (3.62-4.77)	1.31 (1.12-1.52)	<.001	304 (39.84)	2.02 (1.67-2.37)	1.11 (0.91-1.35)	.32	142 (37.87)	1.02 (0.77-1.28)	0.97 (0.74-1.28)	.84
College graduate	348 (24.72)	2.43 (2.04-2.82)	1 [Reference]	NA	207 (27.13)	1.34 (1.07-1.60)	1 [Reference]	NA	118 (31.47)	0.76 (0.58-0.94)	1 [Reference]	NA
Cigarette smoking history^f^												
Current	428 (30.40)	8.00 (6.97-9.05)	5.47 (4.56-6.55)	<.001	202 (26.47)	3.42 (2.78-4.07)	4.78 (3.78-6.06)	<.001	88 (23.47)	1.45 (1.04-1.85)	3.18 (2.27-4.46)	<.001
Former	442 (31.39)	5.29 (4.65-5.93)	4.21 (3.56-4.97)	<.001	212 (27.79)	2.27 (1.82-2.71)	3.69 (2.97-4.58)	<.001	121 (32.27)	1.29 (0.94-1.64)	3.24 (2.43-4.31)	<.001
Never	538 (38.21)	1.57 (1.34-1.80)	1 [Reference]	NA	349 (45.74)	0.92 (0.76-1.08)	1 [Reference]	NA	166 (44.27)	0.51 (0.39-0.62)	1 [Reference]	NA
E-cigarette use history^g^												
Current	534 (37.93)	17.34 (15.52-19.17)	7.53 (6.52-8.71)	<.001	327 (42.86)	9.99 (8.42-11.57)	8.23 (6.80-9.96)	<.001	136 (36.27)	4.18 (3.23-5.14)	5.95 (4.55-7.77)	<.001
Former	336 (23.86)	9.69 (8.27-11.11)	3.79 (3.25-4.42)	<.001	187 (24.51)	5.21 (4.20-6.22)	4.54 (3.69-5.59)	<.001	101 (26.93)	2.96 (2.21-3.71)	4.13 (3.13-5.45)	<.001
Never	538 (38.21)	1.85 (1.61-2.09)	1 [Reference]	NA	249 (32.63)	0.70 (0.57-0.84)	1 [Reference]	NA	138 (36.80)	0.41 (0.32-0.51)	1 [Reference]	NA
Smokeless tobacco use^h^												
Current	140 (9.94)	25.82 (20.72-30.92)	10.50 (8.24-13.39)	<.001	111 (14.55)	20.36 (15.56-25.16)	18.85 (14.14-25.13)	<.001	64 (17.07)	11.81 (8.29-15.32)	16.61 (11.63-23.73)	<.001
Former	435 (30.89)	20.55 (17.94-23.17)	7.57 (6.50-8.82)	<.001	221 (28.96)	8.90 (7.56-10.25)	7.35 (5.97-9.04)	<.001	117 (31.20)	4.81 (3.69-5.94)	6.95 (5.26-9.18)	<.001
Never	833 (59.16)	1.86 (1.65-2.08)	1 [Reference]	NA	431 (56.49)	0.80 (0.68-0.92)	1 [Reference]	NA	194 (51.73)	0.38 (0.31-0.46)	1 [Reference]	NA
Other oral product use^i^												
Past 12-mo	166 (11.79)	40.31 (34.85-45.76)	12.71 (10.06-16.06)	<.001	138 (18.09)	31.86 (26.11-37.61)	21.00 (16.23-27.18)	<.001	65 (17.33)	16.45 (11.28-21.62)	12.75 (9.21-17.65)	<.001
None	1241 (88.14)	2.95 (2.70-3.20)	1 [Reference]	NA	624 (81.78)	1.26 (1.11-1.41)	1 [Reference]	NA	309 (82.41)	0.65 (0.55-0.75)	1 [Reference]	NA
Cannabis use												
Past 12 mo	614 (43.61)	2.24 (1.96-2.52)	1.80 (1.57-2.07)	<.001	212 (27.79)	2.85 (2.36-3.34)	1.92 (1.59-2.32)	<.001	91 (24.27)	1.25 (0.94-1.57)	1.37 (1.05-1.78)	.02
None	383 (27.20)	5.70 (4.93-6.47)	1 [Reference]	NA	308 (40.37)	0.98 (0.81-1.16)	1 [Reference]	NA	180 (48.00)	0.60 (0.47-0.73)	1 [Reference]	NA

Abbreviations: aOR, adjusted odds ratio; HS, high school; GED, General Equivalency Degree; NA, not applicable.

^a^ Unweighted number and column percentage. Numbers may not add up to full sample due to missing data. Models with cell sizes less than 10 should be interpreted with caution due to unreliable estimates.

^b^ Row percentage responding affirmatively compared to those responding No.

^c^ Models with demographic exposure variables (sex, age race, ethnicity, income, education) are adjusted for the other sociodemographic variables, and each model of tobacco/cannabis use exposure variables are adjusted for all sociodemographic variables.

^d^ Significance was determined after Bonferroni adjustment for the three reported outcomes (ever use, past 12-month use, past 30-day use) at a threshold *P* < .0167.

^e^ The other category included Asian and other races including multiracial.

^f^ Current smoking is defined as smoking cigarettes everyday or some days and more than 100 lifetime cigarettes; former smoking, having ever smoked more than 100 cigarettes but not within the past 12 months; and never smoking, having never regularly smoked less than 100 lifetime cigarettes.

^g^ Current vaping is defined as using an e-cigarette regularly or fairly regularly every day or some days; former vaping, having ever used an e-cigarette regularly or fairly regularly but not within the past 12 months; and never vaping, having never regularly used an e-cigarette.

^h^ Smokeless tobacco use is inclusive of dip, chewing tobacco, smokeless tobacco pouches, moist snuff, and snus. Current use is defined as using smokeless regularly or fairly regularly every day or some days; former smokeless, having ever used smokeless regularly or fairly regularly but not within the past 12 months; and never smokeless, having never regularly used smokeless tobacco.

^i^ Other oral tobacco products include nontherapeutic gum or lozenges, toothpicks, discs, dissolvables, and tablets.

## Conclusions

This cross-sectional study found that in the US, oral nicotine pouch use was most prevalent among males, older adolescents and young adults, and individuals who use other nicotine and tobacco products and cannabis. We reported higher prevalence for adults (3.34%) ever using pouches than a 2024 analysis of the 2022 Tobacco Use Supplement to the Current Population Survey, which reported 2.9% ever use.^5^ The 2024 National Youth Tobacco Survey (NYTS) found that 2.4% of high school students endorsed pouch use in the past 30 days,^6^ which is higher than reported here. Importantly, the NYTS data are more recent, which may suggest increasing pouch use among adolescents. However, comparability between surveys is limited by differences in survey methods. Other limitations of this study include the inability to draw longitudinal conclusions (as this was the first PATH wave to measure pouch use), and some results should be interpreted with caution due to small cell sizes. Continued, rigorous surveillance of nicotine pouch use is critical, as is research to understand reasons for pouch use and health outcomes, as the tobacco marketplace evolves.^1^

## Notes

Supplement 1.
eMethods

Supplement 2.
Data Sharing Statement
