# Supplementary material for: Nicotine Pouch Use in Youths and Adults Who Use Cigarettes, E-Cigarettes, and Smokeless Tobacco
Source: JAMA Netw Open. 2025 May 12;8(5):e2511630. doi: 10.1001/jamanetworkopen.2025.11630 (PMC12070233; doi:10.1001/jamanetworkopen.2025.11630)
Supplement: Supplement 1. — eMethods [file jamanetwopen-e2511630-s001.pdf]

Supplemental Online Content

Palmer AM, Smith TT, Chen AA, Rojewski AM, Carpenter MJ, Toll BA. Nicotine pouch use in youths and adults who use cigarettes, e-cigarettes, and smokeless tobacco. *JAMA Netw Open*. 2025;8(5):e2511630. doi:10.1001/jamanetworkopen.2025.11630

eMethods.

This supplemental material has been provided by the authors to give readers additional information about their work.

© 2025 Palmer AM et al. *JAMA Network Open*.

1. Youth/Adolescent Sample Demographics and Characteristics

	Characteristic	n	Weighted % (95% CI)
**Overall**	N=10,632		
**Sex**	**Male**	5553	51.25 (51.18-51.33)
	**Female**	5033	48.74 (48.67-48.82)
**Age group**	**12-14**	4857	51.03 (50.99-51.07)
	**15-17**	5775	48.97 (48.93-49.01)
**Race**	**Black**	1456	15.51 (15.16-15.86)
	**White**	6799	65.25 (64.71-65.80)
	**Other**	1784	19.24 (18.70-19.78)
**Ethnicity**	**Hispanic**	3092	25.82 (25.68-25.95)
	**Non-Hispanic**	7218	74.18 (74.05-74.32)
**Income**	**<$50K**	3673	34.52 (33.12-35.92)
	**$50K+**	6434	65.48 (64.08-66.88)
**Cigarette Smoking History**	**Current**	130	1.09 (0.89-1.30)
	**Former**	598	5.30 (4.82-5.78)
	**Never**	9860	93.61 (93.09-94.12)
**E-cigarette Use History**	**Current**	637	5.44 (5.03-5.85)
	**Former**	1027	9.03 (8.46-9.60)
	**Never**	8897	85.53 (84.83-86.22)
**Smokeless Tobacco Use**	**Current**	21	0.15 (0.07-0.22)
	**Former**	143	1.23 (0.95-1.51)
	**Never**	10468	98.62 (98.34-98.90)
**Other Oral Product Use**	**Past 12-month**	47	0.46 (0.32-0.59)
	**None**	10543	99.54 (99.40-99.68)
**Cannabis Use**	**Past 12-month**	562	6.38 (5.76-6.99)
	**None**	7816	93.62 (93.00-94.24)

© 2025 Palmer AM et al. *JAMA Network Open*.

2. Adult Sample Demographics and Characteristics

	Characteristic	n	Weighted % (95% CI)
**Overall**	N=29,754		
**Sex**	**Male**	14049	48.48 (48.44-48.51)
	**Female**	15661	51.53 (51.49-51.56)
**Age group**	**18-24**	10301	11.65 (11.64-11.65)
	**25-34**	6944	17.34 (16.97-17.71)
	**35-44**	3545	16.91 (16.53-17.28)
	**45-54**	2847	15.71 (15.10-16.31)
	**55-64**	2981	16.84 (16.23-17.45)
	**65 or older**	3134	21.57 (21.55-21.59)
**Race**	**Black**	5163	12.47 (12.26-12.69)
	**White**	19835	74.40 (74.07-74.74)
	**Other**	3600	13.12 (12.89-13.35)
**Ethnicity**	**Hispanic**	6748	17.14 (17.08-17.21)
	**Non-Hispanic**	22478	82.86 (82.79-82.92)
**Income**	**<$50K**	14484	44.47 (43.57-45.37)
	**$50K+**	13531	55.53 (54.63-56.43)
**Education**	**Less than HS**	2971	9.31 (9.01-9.62)
	**HS or GED**	8731	27.99 (27.70-28.29)
	**Some college**	10301	29.70 (29.65-29.75)
	**College grad**	7636	32.99 (32.95-33.04)
**Cigarette Smoking History**	**Current**	5159	13.09 (12.60-13.58)
	**Former**	5967	24.90 (23.97-25.83)
	**Never**	18628	62.01 (60.92-63.09)
**E-cigarette Use History**	**Current**	3139	5.87 (5.60-6.13)
	**Former**	3475	7.44 (7.10-7.79)
	**Never**	23140	86.69 (86.23-87.15)
**Smokeless Tobacco Use**	**Current**	473	1.57 (1.37-1.76)
	**Former**	1747	5.89 (5.52-6.27)
	**Never**	27534	92.54 (92.06-93.02)
**Other Oral Product Use**	**Past 12-month**	408	1.02 (0.88-1.17)
	**None**	29311	98.98 (98.83-99.12)
**Cannabis Use**	**Past 12-month**	5701	16.33 (15.54-17.11)

© 2025 Palmer AM et al. *JAMA Network Open*.

	**None**	19653	83.67<br>(82.89-84.46)

© 2025 Palmer AM et al. *JAMA Network Open*.

3. Survey Items

Note: PATH questionnaires and data are publicly available, with further details provided (https://doi.org/10.3886/ICPSR36498.v22). For all below items, responses of “Don’t know” and “refused” were coded as missing in the present analyses.

1) Age
   a) Age for youth was verified by parent or age derived from emancipated youth's birthdate.
   b) Age for adults was derived from birthdate.

2) Sex
   a) Youth and adults self-reported sex.

What is your sex?	
1	Male
2	Female
	DON'T KNOW
	REFUSED

3) Race

   a) Responses 3-14 were coded as “Other” in the final public dataset.
   b) **Note:** Race and Ethnicity were included in the PATH study to ensure a representative sample, and to allow for analyses of differences in tobacco use and health outcomes (see Hyland et al., doi: 10.1136/tobaccocontrol-2016-052934).

What is your race? Choose all that apply.	
1	White
2	Black or African American
3	American Indian or Alaska Native
4	Asian Indian
5	Chinese
6	Filipino
7	Japanese
8	Korean
9	Vietnamese
10	Other Asian
11	Native Hawaiian
12	Guamanian or Chamorro
13	Samoan
14	Other Pacific Islander
	DON'T KNOW
	REFUSED

© 2025 Palmer AM et al. *JAMA Network Open*.

4) Ethnicity
   a) Responses 2-5 were coded as “Hispanic” in the final public dataset.

Are you Hispanic, [Latino/Latina/Latino or Latina], or of Spanish origin? Choose all that apply.	
1	No, not of Hispanic, [Latino/Latina/Latino or Latina], or Spanish origin
2	Yes, Mexican, Mexican American, [Chicano/Chicana/Chicano or Chicana]
3	Yes, Puerto Rican
4	Yes, Cuban
5	Yes, another Hispanic, [Latino/Latina/Latino or Latina], or Spanish origin
	DON'T KNOW
	REFUSED

5) Education
   a) Adults responded to this item.
   b) In the present analyses, responses 1-2 were coded as “Less than high school (HS),” Reponses 3-4 were coded as “HS or GED,” responses 5-7 were coded as “Some college,” and responses 8-11 were coded as “College Grad.”

What is the highest grade or level of school you have completed?	
1	Less than high school
2	Some high school, no diploma
3	GED
4	High school graduate-diploma
5	Some college but no degree
6	Associate degree-occupational/vocational
7	Associate degree-academic program
8	Bachelor's degree (ex: BA, AB, BS)
9	Master's degree (ex: MA, MS, MEng, MEd, MSW)
10	Professional school degree (ex: MD, DDS, DVM, JD)
11	Doctorate degree (ex: PhD, EdD)
	DON'T KNOW
	REFUSED

6) Income
   a) Parents answered this question for youth/adolescents.
   b) Adults answered this question.
   c) All participants were provided with this orientation text: *This is the total income before taxes of all persons in your household combined. Please include money from jobs, relatives, pensions, dividends, interest, social security payments or retirement benefits, net income from business, farm or rent, and any other money received by household members.*
   d) Responses 1-5 were coded as “<$50k” and responses 6-10 were coded as “≥$50k+.”

Which of the following categories best describes your total **household** income in the past 12 months?	
1	Less than $10,000
2	$10,000 to $14,999
3	$15,000 to $24,999

© 2025 Palmer AM et al. *JAMA Network Open*.

4	$25,000 to $34,999
5	$35,000 to $49,999
6	$50,000 to $74,999
47	$75,000 to $99,999
8	$100,000 to $149,999
9	$150,000 to $199,999
10	$200,000 or more
	DON'T KNOW
	REFUSED

7) Cigarettes
   a) For youth/adolescents in the present analyses, current smoking was defined as having smoking a cigarette within the past 30 days, former smoking is defined as having ever smoked a cigarette but not within the past 30 days, and never smoking is defined as having never smoked a cigarette
   b) For adults in the present analyses, current smoking is defined as smoking cigarettes everyday or some days and >100 lifetime cigarettes, former smoking is defined as having ever smoked >100 cigarettes but not within the past 12 months, and never smoking is defined as having never regularly smoked <100 lifetime cigarettes

Have you ever tried cigarette smoking, even one or two puffs?	
1	Yes
2	No
	DON'T KNOW
	REFUSED

How many cigarettes have you smoked in your entire life? A pack usually has 20 cigarettes in it.	
1	1 or more puffs but never a whole cigarette
2	1 to 10 cigarettes (about ½ pack total)
3	11 to 20 cigarettes (about ½ pack to 1 pack)
4	21 to 50 cigarettes (more than 1 pack but less than 3 packs)
5	51 to 99 cigarettes (more than 2 ½ packs but less than 5 packs)
6	100 or more cigarettes (5 packs or more)
	DON'T KNOW
	REFUSED

When was the last time you smoked a cigarette, even one or two puffs?	
1	Earlier today
2	Not today but sometime in the past 7 days
3	Not in the past 7 days but sometime in the past 30 days
4	Not in the past 30 days but sometime in the past 6 months
5	Not in the past 6 months but sometime in the past year
6	1 to 4 years ago
7	5 or more years ago
	DON'T KNOW
	REFUSED

© 2025 Palmer AM et al. *JAMA Network Open*.

Do you now smoke cigarettes...?	
1	Every day
2	Some days
3	Not at all
	DON'T KNOW
	REFUSED

8) E-cigarettes
   a) For youth/adolescents in the present analyses, current vaping is defined as using an e-cigarette within the past 30 days, former vaping is defined as having ever used an e-cigarette but not within the past 30 days, and never vaping is defined as having never used an e-cigarette.
   b) For adults in the present analyses, current vaping is defined as using an e-cigarette regularly or fairly regularly every day or some days, former vaping is defined as having ever used an e-cigarette regularly or fairly regularly but not within the past 12 months, and never vaping is defined as having never regularly used an e-cigarette
   c) All participants were provided with the below orientation text and a generic image:
      i) Electronic nicotine products include e-cigarettes, pod devices, vape pens, tank systems, mods, e-cigars, e-pipes, e-hookahs, and hookah pens.) Please do not include marijuana or cannabis when answering the following questions about electronic nicotine products. There will be a separate section later about using marijuana or cannabis.

Have you ever used an electronic nicotine product, even one or two times?	
1	Yes
2	No
	DON'T KNOW
	REFUSED

When was the last time you used an electronic nicotine product, even one or two times?	
1	Earlier today
2	Not today but sometime in the past 7 days
3	Not in the past 7 days but sometime in the past 30 days
4	Not in the past 30 days but sometime in the past 6 months
5	Not in the past 6 months but sometime in the past year
6	1 to 4 years ago
7	5 or more years ago
	DON'T KNOW
	REFUSED

Do you now use electronic nicotine products...?	
1	Every day
2	Some days
3	Not at all
	DON'T KNOW
	REFUSED

© 2025 Palmer AM et al. *JAMA Network Open*.

9) Smokeless Tobacco
   a) For both youth/adolescents and adults in the present study, snus and smokeless tobacco items were combined.
   b) For youth/adolescents in the present analyses, current smokeless use is defined as using smokeless tobacco within the past 30 days, former smokeless is defined as having ever used smokeless but not within the past 30 days, and never smokeless is defined as having never used smokeless tobacco.
   c) For adults in the present analyses, current smokeless use is defined as using smokeless regularly or fairly regularly every day or some days, former smokeless is defined as having ever used smokeless regularly or fairly regularly but not within the past 12 months, and never smokeless is defined as having never regularly used smokeless tobacco.
   d) All participants were provided with the below orientation text and a generic image:
      (1) Snus is a type of smokeless tobacco. Snus usually comes in small pouches, although some snus may be sold as loose snus. Typically, with most kinds of smokeless tobacco, you spit, but you do not need to spit when using snus. Common brands of snus include Camel Snus, Marlboro Snus, and General Snus. Please think only about snus as you answer the following questions. There will be a separate section later about other types of smokeless tobacco products (such as moist snuff, dip, spit, or chew), nicotine pouches (such as Zyn, on!, and Velo), and other types of oral nicotine products.
      (2) The next questions are about smokeless tobacco other than snus, nicotine pouches, or other types of oral nicotine products. Smokeless tobacco is a type of tobacco which you put in your mouth and chew, suck, or spit. It may be loose or packaged in small pouches that you can place directly in your mouth. There are many kinds of smokeless tobacco, such as dip, spit, moist snuff, pouches, and chewing tobacco.

Have you ever used snus, even one or two times?	
1	Yes
2	No
	DON'T KNOW
	REFUSED

When did you last use snus even one or two times?	
1	Earlier today
2	Not today but sometime in the past 7 days
3	Not in the past 7 days but sometime in the past 30 days
4	Not in the past 30 days but sometime in the past 6 months
5	Not in the past 6 months but sometime in the past year
6	1 to 4 years ago
7	5 or more years ago
	DON'T KNOW
	REFUSED

Do you now use snus...?	
1	Every day
2	Some days
3	Not at all
	DON'T KNOW
	REFUSED

© 2025 Palmer AM et al. *JAMA Network Open*.

Have you ever used smokeless tobacco, such as dip, spit, moist snuff, pouches, or chewing tobacco, even one or two times?	
1	Yes
2	No
	DON'T KNOW
	REFUSED

When did you last use smokeless tobacco, even one or two times?	
1	Earlier today
2	Not today but sometime in the past 7 days
3	Not in the past 7 days but sometime in the past 30 days
4	Not in the past 30 days but sometime in the past 6 months
5	Not in the past 6 months but sometime in the past year
6	1 to 4 years ago
7	5 or more years ago
	DON'T KNOW
	REFUSED

Do you now use smokeless tobacco...?	
1	Every day
2	Some days
3	Not at all
	DON'T KNOW
	REFUSED

10) Oral nicotine Products
    a) Youth/Adolescents and adults were asked this question.
    b) All participants were provided with the below orientation text and a generic image:
        i) *The next questions are about types of oral nicotine products that you put in your mouth, other than nicotine pouches. This includes lozenges, discs, tablets, gum, toothpicks, dissolvable tobacco, and related products. Common brands include Velo Nicotine Lozenges, Verve Nicotine Discs, Rogue Nicotine Tablets, Lucy Nicotine Gum, Pixotine Nicotine Toothpicks, and Stonewall Dissolvable Tobacco. Please do* ***not*** *include nicotine replacement therapy products (such as Nicorette lozenges, Nicorette gum, or NicoDerm patches) when answering the following questions.*

In the past 12 months, have you used an oral nicotine product, even one or two times?	
1	Yes
2	No
	DON'T KNOW
	REFUSED

11) Cannabis.

    a) Youth/Adolescents and adults were asked this question.

In the past 12 months, have you used marijuana?	
1	Yes
2	No
	DON'T KNOW
	REFUSED

© 2025 Palmer AM et al. *JAMA Network Open*.

12) Nicotine Pouches

a) Youth/Adolescents and adults were asked these questions.
b) All participants were provided with the below orientation text and a generic image:
   i) The next questions ask about small, white pouches that contain nicotine which users place in their mouth. ***Nicotine pouches*** *are different from other smokeless tobacco products such as snus, dip, or chew, because they do not contain any ground tobacco leaf. Common brands include Zyn, on!, or Velo, but there are many others.*

Have you ever used nicotine pouches, even one or two times?	
1	Yes
2	No
	DON'T KNOW
	REFUSED

In the past 12 months, have you used nicotine pouches, even one or two times?	
1	Yes
2	No
	DON'T KNOW
	REFUSED

In the past 30 days, have you used nicotine pouches, even one or two times?	
1	Yes
2	No
	DON'T KNOW
	REFUSED

© 2025 Palmer AM et al. *JAMA Network Open*.
