# Supplementary material for: Nicotine Pouch Use in Youths and Adults Who Use Cigarettes, E-Cigarettes, and Smokeless Tobacco
Source: JAMA Netw Open. 2025 May 12;8(5):e2511630. doi: 10.1001/jamanetworkopen.2025.11630 (PMC12070233; doi:10.1001/jamanetworkopen.2025.11630)
Supplement: Supplement 2. — Data Sharing Statement [file jamanetwopen-e2511630-s002.pdf]

Data Sharing Statement

Palmer. Nicotine Pouch Use in Youths and Adults Who Use Cigarettes, E-Cigarettes, and Smokeless Tobacco. *JAMA Netw Open*. Published May 12, 2025.
doi:10.1001/jamanetworkopen.2025.11630

Data
Data available: Yes
Data types: Deidentified participant data, Data dictionary
How to access data: https://www.icpsr.umich.edu/web/NAHDAP/studies/36498/
When available: With publication

Supporting Documents
Document types: None

Additional Information
Who can access the data: Data are publicly available to those who register with and agree to terms of use with ICPSR
Types of analyses: Data are publicly available to those who register with and agree to terms of use with ICPSR
Mechanisms of data availability: Data are publicly available to those who register with and agree to terms of use with ICPSR
